# Physics-Enhanced Orthogonal Sensing for Self-Supervised Anomaly Detection in Rolling Mills

**DOI:** 10.3390/s26092895

**Published:** 2026-05-05

**Authors:** Yifan Wang, Bin Zheng, Yehan Feng, Xiong Chen

**Affiliations:** 1 School of Information Science and Technology, Fudan University, Shanghai 200438, China; 2 Business Department for Quality Special Steels, Guangdong Zhongnan Iron and Steel Co., Ltd., Shaoguan 512100, China

**Keywords:** rolling mill, guide system, anomaly detection, self-supervised learning, orthogonal sensing, CSD transformer, VQ-VAE, condition monitoring

## Abstract

The rolling mill guiding system is a key component that affects the quality of steel products. However, due to the harsh on-site environment, there is usually a lack of effective online monitoring and early warning mechanisms. Moreover, in industrial environments, fault samples are very scarce, making supervised artificial intelligence methods difficult to apply. This paper proposes a “physics-enhanced” orthogonal-sensing cyber-physical architecture that integrates hardware and software design. At the hardware level, an embedded orthogonal sensing layout (P⊥V) is designed to decouple drive-chain vibration from rolling-force fluctuations at the transducer level. At the algorithm level, the state monitoring of the guiding system is formulated as a self-supervised anomaly detection problem, and a two-branch network architecture is designed: one branch uses the CSD transformer to capture physical coupling characteristics, while the other branch uses VQ-VAE to extract operating-condition context. Experimental results on a dataset comprising real operational data and expert-validated synthetic fault scenarios show that the system achieves an AUC-ROC of 0.952 and a false alarm rate of 0.048 under a 95% TPR, with an end-to-end processing latency of approximately 8 ms per window and a system-level fault response time of approximately 108 ms, and thus meets the requirements of real-time industrial monitoring.

## 1. Introduction

The trend of Industry 4.0 has fundamentally transformed the steel manufacturing industry [1,2]. Smart sensors and data-based decision-making processes have gradually permeated every stage of steel production. Although a great deal of research has been conducted on the health management of major equipment such as main drive units of rolling mills and backup rolls [3,4], an important type of component, the roller guides, has received relatively little attention. The roller guides come into direct contact with the hot steel billets during each rolling pass and are the first line of defense for reducing surface defects of the steel. In this sense, the roller guides are among the components most directly related to product quality, and intelligent condition monitoring and early warning for this component are therefore critical.

Currently, the condition assessment of the roller guide system mainly relies on regular manual inspections [5,6]. However, this approach has obvious limitations: workers need to be close to the high-temperature and high-speed production lines, facing potential risks of accidents such as flying steel; at the same time, the frequency of manual inspections is limited and there is a significant lag. Therefore, embedding sensors in the roller guide system and achieving intelligent monitoring is an urgent need to improve monitoring efficiency and safety.

However, achieving sensor intelligence for the roller guide system faces two core challenges [3,7]. The first challenge lies in the hardware aspect: the extreme environment in the hot rolling site makes most conventional sensors prone to damage, which requires ingenious design in the selection of sensors and their installation positions. The second challenge lies in the algorithmic aspect: even if sensor data is successfully obtained, the data itself has two characteristics that make conventional supervised learning ineffective. The first is severe non-stationarity [8]: during actual production, it will rapidly go through different stages such as idle, biting steel, steady-state rolling, and tailing, with fluctuations in rolling force and steel billet temperature causing complex distributional changes in sensor signals, which no single fixed model can handle. The second is that most of the data is unlabeled [9]. Due to the extremely high cost of guide system failures, on-site workers often intervene in advance, and the production line is usually in a normal production state, resulting in the lack of training labels for supervised learning. Although self-supervised learning (SSL) can bypass label dependence [10,11], the existing SSL methods mainly perform unconditional density estimation. While recent self-supervised methods such as TS2Vec [12] and Anomaly Transformer [13] excel at capturing temporal dependencies, they process all modalities symmetrically and optimize for sequence-level invariance. This symmetric treatment fails to capture the inherent physical causal asymmetry under non-stationary industrial regimes. Our proposed framework bridges this gap by explicitly treating the excitation modality as a contextual condition rather than a symmetric feature.

**Figure 1:**
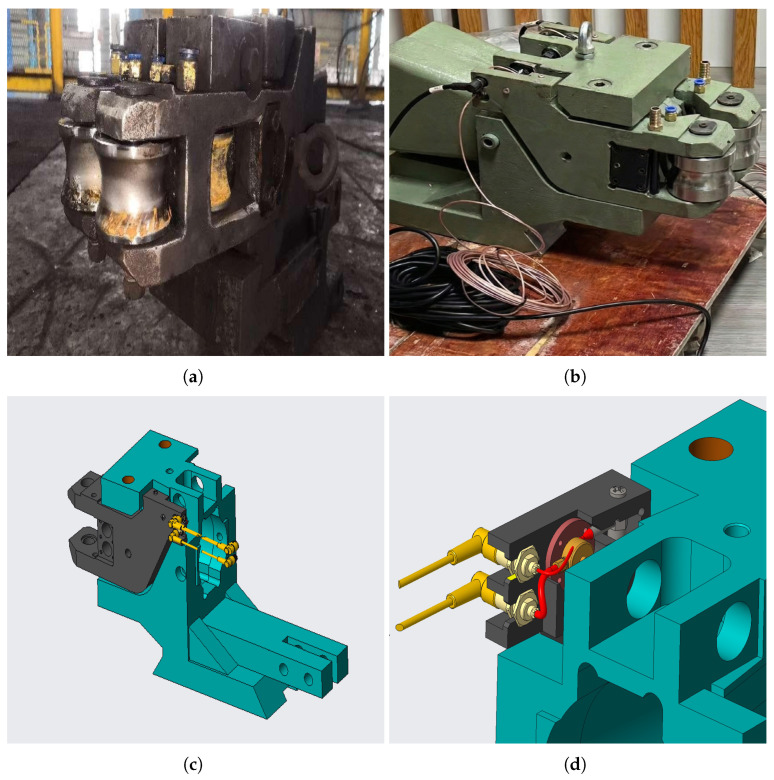
From traditional to intelligent roller guide. (**a**) Conventional roller guide deployed on the hot rolling production line, with no integrated sensing capability. (**b**) Intelligent roller guide with embedded sensors and shielded signal cables, assembled in the workshop prior to installation. (**c**) CAD model of the intelligent guide design, showing the machined sensor mounting provisions in the guide-arm body. (**d**) Close-up view of the sensor installation bay, where the red part denotes the pressure-sensor base, the yellow part denotes the pressure sensor, and the gray part denotes the vibration sensor.

In response to the challenges in both hardware and algorithms, this paper proposes an “orthogonal physics-enhanced” cyber-physical architecture based on hardware–software collaboration. At the hardware level, we embedded an orthogonal sensing layout (P⊥V) on each guide arm. Through the physical orthogonal installation of pressure sensors and vibration sensors, we achieved decoupling of clamping force and structural vibration at the signal source [14]. At the algorithm level, we designed a dual-branch self-supervised network: the Coupling Branch constructs a 16×16 cross-spectral density (CSD) matrix [15] from virtually expanded channels and models the physical coupling patterns between sensors using the CSD Pair-Token Transformer; the Context Branch discretizes the pressure trajectory into working condition prototypes using a dual-channel VQ-VAE [16] and generates conditional context vectors. The two branches collaborate through a physics-context gated fusion mechanism, achieving a transition from unconditional to conditional density estimation, which makes the anomaly score remain sensitive to coupling anomalies and at the same time keeps robustness to normal working condition changes. Figure 1 shows the comparison between traditional guide arms and intelligent guide arms, as well as the sensor integration and orthogonal sensing layout based on the minimally invasive principle.

The main contributions of this paper are summarized as follows:

- **Engineering Innovation.** We designed and deployed an embedded orthogonal sensing system for the roller guide system, addressing the gap in intelligent monitoring hardware for such critical but previously overlooked components of the rolling machine. Unlike existing commercial solutions that focus on electric adjustment or simple threshold alarms, our design emphasizes low cost and high fidelity state perception, and reaches built-in decoupling of physical signals through the P⊥V layout. Due to spatial constraints and the mechanical necessity of preserving guide structural integrity, non-orthogonal or isolated single-sensor configurations are physically prohibitive. However, our ablation studies empirically confirm that the multi-modal orthogonal integration is critical to preventing single-modality fault masking.
- **Algorithm Innovation.** We proposed a dual-branch self-supervised anomaly detection architecture without any fault labels. The Coupling Branch captures the physical coupling patterns between sensors from the cross-spectral density matrix through the CSD Pair-Token Transformer; the Context Branch extracts discrete working condition prototypes through VQ-VAE. The main novelty lies in integrating these components into a self-supervised objective that explicitly embeds the physical causal prior of load excitation and structural response, thereby transforming conventional feature distances into a physics-consistency measure.
- **Practical Significance.** Experimental evaluation on a real operating hot rolling production line shows that the system’s AUC-ROC reaches 0.952, the FPR@95%TPR is 0.048, and the false positive rate at the 95% TPR operating point is reduced from 0.062 to 0.048 compared with the strongest baseline. The end-to-end processing latency per window is approximately 8 ms, and the system-level fault response time is approximately 108 ms, meeting the requirements of real-time industrial monitoring.

## 2. Related Work

### 2.1. Guide System Monitoring in Rolling Mills

In industry, some rolling mill equipment suppliers, such as Primetals Technologies and Danieli, have developed automated roller guide modules equipped with servo-driven gap adjustment and built-in monitoring functions. However, these commercial solutions still have several limitations related to this study. First, their monitoring relies on single-sensor statistical indicators, which can only monitor fault information in a single dimension and cannot utilize the cross-modal coupling relationship between force and vibration channels. Second, the underlying signal processing logic is proprietary and not publicly available, making independent algorithm improvements or adjustments for specific production lines extremely difficult. Third, there are no publicly available commercial systems that can perform the self-supervised, physics-based cross-spectral anomaly detection proposed in this study.

Patents tell a similar story. The existing patents related to roller guide technology are almost all focused on improvements in the mechanical structure. For instance, one patent [17] describes a quick-change structure to cut roller replacement downtime; another [18] proposes a new type of wear-resistant material combination for guide rollers, aiming to extend the service life under extreme high temperatures and wear conditions. Although these innovations are of great value for improving operational efficiency and extending component lifespans, they all lack intelligent sensing devices and algorithms for fault monitoring.

In short, few roller guide systems combine structurally integrated multimodal sensing with a fault monitoring algorithm that explicitly exploits cross-sensor coupling.

### 2.2. Self-Supervised Learning for Industrial Anomaly Detection

As mentioned in Section 1, the natural scarcity of labels in industrial monitoring makes it difficult to implement supervised methods [9,19], pushing the field toward unsupervised or self-supervised learning solutions.

Traditional unsupervised anomaly detection methods attempt to address the problem of the lack of labels in the training set by learning a compact description of normal behavior. Support Vector Data Description (SVDD) [20] maps normal samples into the smallest-volume hypersphere within the feature space, and marks points outside the boundary as anomalies; its extension, Deep SVDD [21], learns the feature mapping end-to-end through a neural network. Methods based on variational autoencoders [22] also model normality through reconstruction probability, and classify samples with low reconstruction probability as anomalies. These methods perform well on low-dimensional or steady-state benchmark datasets, but due to the “normal” distribution in high-dimensional, multimodal industrial time series being non-stationary and context-dependent [23], their performance is poor.

Self-supervised learning (SSL) extracts transferable representations from unlabeled data by solving carefully designed pretext tasks. Zhang et al. [24] systematically classified the time series SSL methods, and they can mainly be divided into three categories:

1. Contrastive methods, such as Contrastive Predictive Coding (CPC) [10], SimCLR [11], and Momentum Contrast (MoCo) [25], learn representations by bringing together semantically similar positive sample pairs and pushing apart dissimilar negative sample pairs, typically using the InfoNCE objective function.
2. Masked reconstruction methods, inspired by BERT’s masked language modeling [26], were later extended to the visual domain by Masked Autoencoders (MAE) [27], training the model to predict the missing parts of the input from the context that was not masked.
3. Discrete representation methods, represented by VQ-VAE [16], learn a finite prototype vector codebook, encoding each input fragment into its nearest codebook entry, generating a discrete latent space that clusters the running states into a limited number of typical states.

Recent studies have begun to apply these SSL paradigms to industrial fault diagnosis and anomaly detection, each addressing some issues but none of them fully solved the problem. Contrastive methods have shown that the representations learned from unlabeled time series can be comparable to those of supervised features. TS-TCC [28] and T-Loss [29] conduct contrastive learning on time series using time-augmented windows for downstream bearing and gearbox classification; TS2Vec [12] demonstrates that hierarchical contrastive learning can generate cross-domain universal embeddings. CAROTS [30] further introduces causal relationships through causality-preserving and causality-disturbing augmentations. This partially captures the structure between variables, but still does not clearly model conditions based on the operating state. However, these contrastive objectives optimise for invariance to augmentations rather than detecting subtle deviations in the cross-spectral coupling structure of the orthogonal sensing system—and this deviation is exactly the earliest fault feature. Masked reconstruction and association-discrepancy methods use different approaches. Anomaly Transformer [13] detects anomalies through learned association discrepancy, and does not require labeled abnormal samples during training. DCdetector [31] further eliminates the reliance on explicit negative samples by working through a dual-branch attention map constructed from the original query and permuted queries. Although these methods effectively capture anomalies in the time dimension, they symmetrically process all input channels and ignore the causal asymmetry between excitation and response that naturally exists in the cyber-physical sensor configuration. General time series backbone networks such as TimesNet [32] (which reshapes one-dimensional sequences into two-dimensional tensors through periodic folding) and DACR [33] (which improves robustness under distribution perturbations through enhanced latent distributions) provide powerful feature extraction capabilities, but are not sensitive to the physical characteristics of the monitored system.

Although all these frameworks have their strengths, they do not take into account the changing operating conditions over time when estimating density. Therefore, robustness under real industrial non-stationarity remains an open problem.

Kim et al. [34] demonstrated that Reversible Instance Normalization (RevIN) can alleviate the distribution shift in time series prediction by first removing and then restoring the instance-level statistics. However, as Liu et al. [35] pointed out, excessive normalization may erase the amplitude information with physical significance; this information can distinguish between benign state transitions and initial failures. A model that normalizes without considering the running context fundamentally cannot evaluate the normality that depends on the context. Domain adaptation methods [36] operate at the domain level rather than the sample level, which makes them less suitable for rapid operational fluctuations in the hot rolling process. Therefore, a conditional framework is needed, using pressure as the context condition for vibration modeling, Pr(vibrationanomaly∣pressurecontext), rather than simply modeling Pr(anomaly).

These unresolved common issues have driven us to propose a dual-branch, context-conditioned architecture in Section 3.

## 3. Intelligent Guide System and Problem Formulation

This section introduces the intelligent roller guide platform and formally defines the anomaly detection problem.

### 3.1. Hardware Implementation: From Traditional to Intelligent

In bar and wire rod rolling mills, the traditional guide system consists of purely mechanical components, including a guide box, a pair of guide arms, roller shafts, and adjusting screws. Although they can perform the basic functions of centering and stabilizing the steel billet in each rolling pass, they do not possess any sensing or diagnostic capabilities. Therefore, the assessment of the guide status is entirely dependent on regular manual inspections, and preventive replacement measures are taken, which increases production costs and often fails to detect problems in a timely manner. This study addresses this issue by installing an embedded multimodal sensing system on the traditional guides, transforming them into a cyber-physical component that can autonomously and continuously collect data during steel production (Figure 1a,b).

#### 3.1.1. Retrofit Design and Sensor Selection

After conducting a detailed structural assessment and extensive communication with on-site operators and domain experts, based on the “minimally invasive” design principle, we directly embedded the sensors within the main body of the guiding mechanism, maintaining its original structure and rigidity (Figure 1). Each guide arm rotates around the support pin and functions as a lever mechanism: the rollers at the distal end directly contact the steel billet, generating clamping force and vibration, which are transmitted along the arm body to the proximal pivot area. This mechanical structure is of great significance for sensing: any degradation of the guiding function (whether it is offset, bearing wear of the rollers, or structural fatigue) will change the magnitude and direction of the transmitted force, and these changes are expected to be reflected by the mechanical response measured along the arm body. The installation of the sensors takes advantage of this principle, after precisely machining the arm body in the low-stress areas (Figure 1c,d), the sensors are embedded therein. While exhaustive destructive or long-term fatigue testing data remains proprietary, the functional integrity of the instrumented guide was validated through its continuous operation on an active production line for several months. During this extended period, the production of qualified steel met all metallurgical and dimensional standards verified by domain experts, confirming that the internal sensor machining did not materially compromise structural rigidity or operational safety. This embedded layout ensures the mechanical connection of the force flow network between the sensor and the arm body, while avoiding direct contact with the high-temperature steel billet and high-pressure cooling water. This design achieves effective state sensing while having the lowest actual cost and structural changes. The sensor module is installed using a snap-fit installation mechanism, and can be quickly replaced during the planned machine downtime.

**Figure 2:**
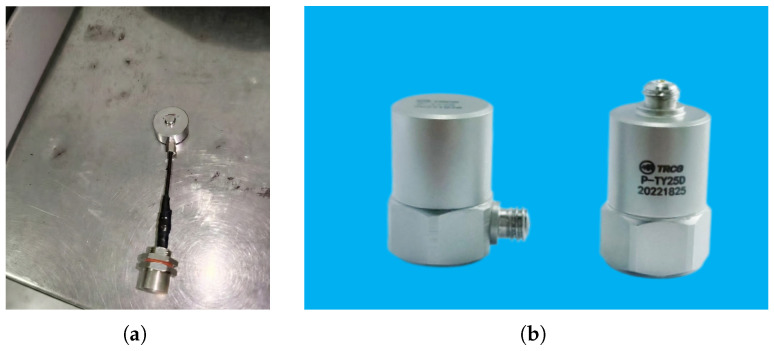
Physical appearance of the two sensor types deployed on each intelligent roller guide arm. (**a**) F1005-20000 resistive pressure sensor with shielded cable and IP67 aviation connector. (**b**) WKD0181 charge-output piezoelectric accelerometers in hermetic stainless-steel housing.

Each guide arm is equipped with an F1005-20000 resistive strain-gauge pressure sensor (Senther Technology, Shenzhen, China) ($P_{1}$, $P_{2}$) for measuring clamping force, and a WKD0181 piezoelectric accelerometer (Tianjin Weekend Measurement and Control Equipment Technology Co., Ltd., Tianjin, China) ($V_{1}$, $V_{2}$) for vibration monitoring. The sensing axis of the accelerometer is perpendicular to the corresponding pressure sensor. Table 1 summarizes the detailed specifications, and the appearance of these two sensors is shown in Figure 2.

#### 3.1.2. Orthogonal Sensing Configuration

One of the key features of this sensing system lies in the arrangement of the pressure sensor and the vibration sensor on each guide arm in a physically orthogonal manner, labeled as P⊥V. The pressure sensor is placed along the clamping-force direction, which is along the axis of the main rolling load; the accelerometer detects the lateral vibration response. This layout takes advantage of a fundamental physical asymmetry in the guiding system: the pressure channel captures the excitation, while the vibration channel captures the structural response caused by this excitation. From the perspective of structural dynamics, this excitation–response relationship can be represented in the frequency domain by the mechanical transfer function [14]:

(1) V(ω)=H(ω)·P(ω)+N(ω),

where H(ω) is the frequency-dependent transfer function of the guide arm structure, and N(ω) is the measurement noise independent of the excitation. Under normal operating conditions, H(ω) remains stable during continuous rolling: pressure fluctuations during head impact and load pulsation are transmitted to the vibration channel with a determined amplitude ratio and phase delay. Therefore, the cross-spectral density (CSD) between *P* and *V* shows limited coherence, with its range being confined within predictable physical frequency bands [15].

**Figure 3:**
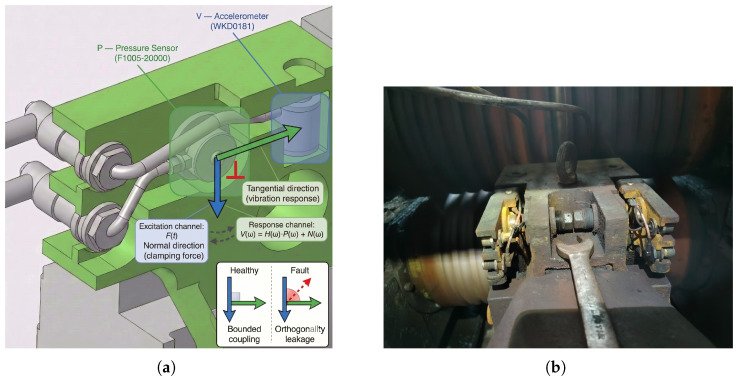
Orthogonal sensing layout (P⊥V) on each roller guide arm. (**a**) Annotated CAD model with sensing directions, where the blue arrow indicates the vibration-sensor direction and the green arrow indicates the pressure-sensor direction. The red right-angle marker denotes the orthogonal arrangement, and the inset illustrates bounded coupling under healthy operation and orthogonality leakage under faults. (**b**) Field photograph of the deployed instrumented guide.

**Figure 4:**
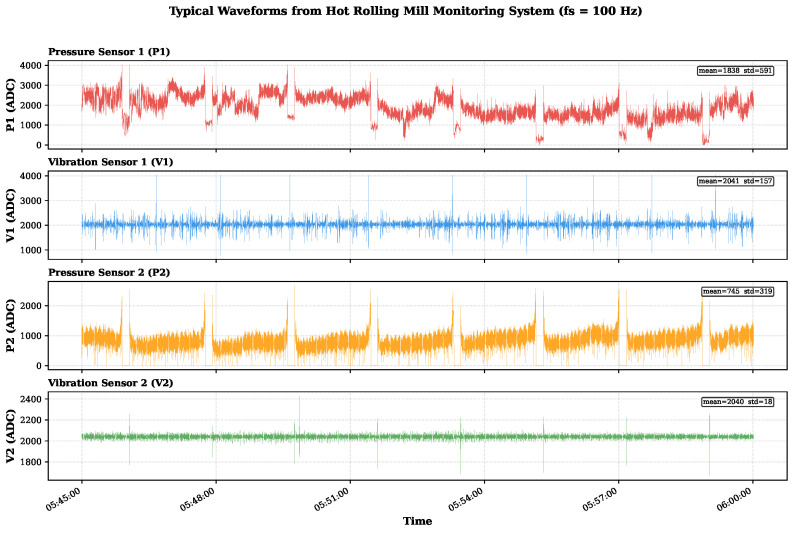
Representative four-channel signal waveforms recorded during continuous production rolling. The four panels correspond to $P_{1}$, $V_{1}$, $P_{2}$, and $V_{2}$, respectively.

When a fault occurs, the physical orthogonality is disrupted, leading to abnormal energy transfer between the two sensing axes. This is manifested as an increase in the off-diagonal elements of the CSD matrix, thereby forming abnormal features based on physical principles. These features cannot be detected by single-modal measurements. Figure 3 shows the arrangement of the orthogonal sensors. Figure 4 shows representative four-channel waveforms collected under production conditions, showing non-stationary phase changes.

### 3.2. Mathematical Formulation

#### 3.2.1. Problem Statement

Let $D_{\mathrm{train}}={\left\{X_{i}\right\}}_{i=1}^{N}$ denote a training set comprising *N* fixed-length observation windows collected exclusively under normal operating conditions. Each observation window $X_{i}$ encompasses synchronised segments from all four sensor channels:

(2) $$X_{i}={\left\{P_{1}^{\left(i\right)}\left(t\right),\;V_{1}^{\left(i\right)}\left(t\right),\;P_{2}^{\left(i\right)}\left(t\right),\;V_{2}^{\left(i\right)}\left(t\right)\right\}}_{t=1}^{T},$$

where *T* is the window length in samples. The objective is to learn a parametric model $p_{\theta }\left(X\right)$ of the joint distribution over normal observations, such that at inference time a previously unseen window $X^{*}$ can be scored by how much it deviates from the learned normal distribution.

Because fault labels are unavailable during training, the problem is formulated as unsupervised density estimation for anomaly detection [19,37]. The model is trained exclusively on normal data, and anomalies are defined implicitly as observations that fall in low-density regions of the learned distribution.

#### 3.2.2. Dual-Branch Input Decomposition

We decompose the raw multimodal observation X into two complementary feature branches:

(3) $$X\left(t\right)=\left[X_{\mathrm{coupling}}(t),\;\;X_{\mathrm{context}}\left(t\right)\right].$$

The coupling branch ($X_{\mathrm{coupling}}$) encodes the frequency-domain cross-spectral coupling structure among all sensor channels. The construction goes through three stages: adaptive virtual channel expansion, short-time spectral analysis, and cross-spectral density matrix estimation.

Stage 1: Adaptive virtual channel expansion. Starting from the raw four-channel signal $x\left(t\right)={\left[P_{1}\left(t\right),\;V_{1}\left(t\right),\;P_{2}\left(t\right),\;V_{2}\left(t\right)\right]}^{\;⊤}$, each physical channel $x_{c}\left(t\right)$ ($c\in \{P_{1},V_{1},P_{2},V_{2}\}$) is independently decomposed into $2^{L}$ orthogonal sub-bands using a wavelet packet decomposition (WPD) [38] of depth L=3:

(4) $$S^{\left(c\right)}=\left\{s_{1}^{\left(c\right)}\left(t\right),\;s_{2}^{\left(c\right)}\left(t\right),\;\ldots ,\;s_{2^{L}}^{\left(c\right)}\left(t\right)\right\},$$

where each $s_{j}^{\left(c\right)}\left(t\right)$ represents the signal component within the *j*-th frequency sub-band. To select the sub-bands carrying the most diagnostic information, a combined score of impulse sensitivity and spectral complexity is computed for each sub-band:

(5) $${\mathrm{Score}}_{j}^{\left(c\right)}=\underset{\mathrm{impulse}\;\mathrm{sensitivity}}{\underbrace{\mathrm{Kurt}\left(s_{j}^{\left(c\right)}\right)}}+\lambda \cdot \underset{\mathrm{spectral}\;\mathrm{complexity}}{\underbrace{H\left(s_{j}^{\left(c\right)}\right)}},$$

where Kurt(·) denotes excess kurtosis and H(·) is spectral entropy, and λ balances the two criteria. For each physical channel, the top-*K* (K=3) scoring sub-bands are retained alongside the original broadband signal, giving a (1+K)-dimensional expanded representation per channel. The complete network dimensions and hyperparameters are provided in Section 5.2. With L=3 and K=3, the four physical channels are expanded into a 16-dimensional augmented signal vector:

(6) $$x_{16}\left(t\right)={\left[\underset{\mathrm{Arm}\;1\;\mathrm{pressure}\;\mathrm{group}}{\underbrace{P_{1},\;s_{b_{1}}^{\left(P_{1}\right)},\;s_{b_{2}}^{\left(P_{1}\right)},\;s_{b_{3}}^{\left(P_{1}\right)}}},\;\;\underset{\mathrm{Arm}\;1\;\mathrm{vibration}}{\underbrace{V_{1},\;s_{b_{1}}^{\left(V_{1}\right)},\;\ldots }},\;\;\underset{\mathrm{Arm}\;2\;\mathrm{pressure}}{\underbrace{P_{2},\;\ldots }},\;\;\underset{\mathrm{Arm}\;2\;\mathrm{vibration}}{\underbrace{V_{2},\;\ldots }}\right]}^{\;⊤}\;\in R^{16},$$

where $b_{1},b_{2},b_{3}$ denote the indices of the three highest-scoring sub-bands for each channel; the selected indices generally differ across channels.

Stage 2: Short-time Fourier transform. A short-time Fourier transform (STFT) with Hann windowing and 50% overlap is applied to each of the 16 channels following the standard Welch configuration [39,40], yielding complex-valued spectral vectors $X_{16}\left(m,\;k\right)\in C^{16}$ at each time–frequency bin.

Stage 3: Cross-spectral density matrix estimation. The 16×16 cross-spectral density (CSD) matrix at frequency bin *k* is estimated by averaging the outer products of the spectral vectors over the $K_{s}$ segments, following Welch’s method [39]:

(7) $$\hat{M}\left(k\right)=\frac{1}{K_{s}}\sum_{m=0}^{K_{s}-1}X_{16}\left(m,\;k\right)\;X_{16}{\left(m,\;k\right)}^{H}\;\in \;C^{16\times 16},$$

where ${\left(\cdot \right)}^{H}$ denotes the conjugate transpose, and *m* denotes the index of overlapping segments, providing an asymptotically unbiased estimate in accordance with Welch’s method. By construction, $\hat{M}\left(k\right)$ is Hermitian and positive semi-definite; a small diagonal regularisation $\epsilon I_{16}$ ensures strict positive definiteness [14]. The diagonal entries of $\hat{M}\left(k\right)$ encode single-channel auto-spectral power densities, while the off-diagonal entries encode inter-channel coherence and phase relationships. The 4×4 block structure of $\hat{M}\left(k\right)$ directly reflects four physically distinct coupling categories:

- Diagonal blocks (intra-group): cross-frequency modulation within a single physical channel, sensitive to local resonance excitation by bearing or gear defects.
- Same-arm *P*–*V* blocks: cross-modal coupling on one guide arm, directly encoding the P⊥V orthogonality and thus the primary indicator of force–vibration coupling anomalies.
- Cross-arm same-modal blocks ($P_{1}$–$P_{2}$ or $V_{1}$–$V_{2}$): bilateral coordination, sensitive to asymmetric loading, unilateral wear, or misalignment.
- Cross-arm cross-modal blocks: system-level coupling, indicative of global structural anomalies or installation defects.

The complete CSD matrix at each frequency bin is therefore a compact representation based on physical principles of the multi-channel coupling state; its detailed processing by the proposed Transformer backbone is described in Section 4.

The context branch ($X_{\mathrm{context}}$) processes the pressure signals $\left[P_{1}\left(t\right),\;P_{2}\left(t\right)\right]$ in the time domain to extract operating-condition context. The pressure signal is treated as a context encoder for the current loading regime. This design has a physical basis: the absolute pressure level determines the mechanical loading state of the guide-arm system and dictates the vibration patterns that should be considered “normal” under a given regime.

A 1-D convolutional encoder maps the bivariate pressure signal $P\left(t\right)\in R^{T\times 2}$ to a latent sequence $Z_{e}\in R^{T^{\prime }\times d}$, and each latent frame is independently quantised against a learnable codebook $C=\{e_{1},\ldots ,e_{K}\}$ via a Vector Quantised Variational Autoencoder (VQ-VAE) [16]:

(8) $$z_{q}\left(t^{\prime }\right)=e_{k^{*}\left(t^{\prime }\right)},\;k^{*}\left(t^{\prime }\right)=\arg\min_{k}{∥z_{e}\left(t^{\prime }\right)-e_{k}∥}_{2}.$$

The resulting codebook index sequence records the operating-state trajectory. Temporal statistics of the quantised sequence, combined with raw per-channel pressure statistics, are projected through an MLP to produce the context vector $X_{\mathrm{context}}\in R^{d}$ that encodes both the type and magnitude of the prevailing operating load. The context branch thus provides the conditioning signal for context-aware anomaly assessment. The detailed network architecture is presented in Section 4.3.

#### 3.2.3. Conditional Anomaly Scoring

Given the dual-branch decomposition, the joint distribution of normal observations can be factored as

(9) $$p_{\theta }\left(X\right)=p_{\theta }\;\left(X_{\mathrm{coupling}}|X_{\mathrm{context}}\right)\cdot p_{\theta }\;\left(X_{\mathrm{context}}\right).$$

This factorisation reflects the physical causal relationship that naturally exists in the guide system: the operating context (pressure state) determines the expected coupling pattern (vibration structure), and the anomaly assessment should evaluate whether the observed coupling is consistent with the current context. The anomaly score for a test observation $X^{*}$ is accordingly defined as

(10) $$S\left(X^{*}\right)=-\log\;p_{\theta }\;\left(X_{\mathrm{coupling}}^{*}|X_{\mathrm{context}}^{*}\right),$$

where a high score indicates that the observed coupling pattern is physically inconsistent with the current operating context. The marginal context distribution $p_{\theta }\left(X_{\mathrm{context}}\right)$ is excluded from the anomaly score because the pressure modality serves as a conditioning signal rather than an independent anomaly indicator.

## 4. Methodology: Dual-Branch Self-Supervised Architecture

This section introduces the neural network architectures, cross-modal fusion mechanism, and training strategy.

### 4.1. Architecture Overview

The proposed architecture consists of two processing branches and a physics-gated fusion module. The Coupling Branch maps $\hat{M}\left(k\right)$ to a coupling feature $Z_{\mathrm{CSD}}\in R^{d}$; the Context Branch maps $\left[P_{1}\left(t\right),P_{2}\left(t\right)\right]$ to a context vector $Q_{\mathrm{context}}\in R^{d}$. The fusion module generates a context-predicted coupling feature $Z_{\mathrm{expected}}$, and the anomaly score is the deviation $∥Z_{\mathrm{expected}}-Z_{\mathrm{CSD}}∥$ (Equation (10)).

**Figure 5:**
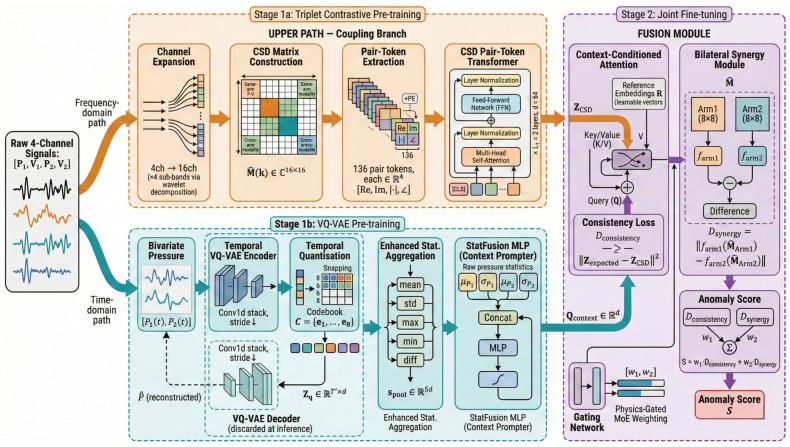
Overall architecture of the proposed dual-branch self-supervised anomaly detection system. Arrows indicate the data flow and training/fusion flow; the orange upper path denotes the Coupling Branch, the cyan lower path denotes the Context/VQ-VAE Branch, and the purple region denotes the fusion and anomaly-scoring module.

The entire system is trained in a two-stage self-supervised pipeline. In Stage 1, the Coupling Branch and the Context Branch are pre-trained independently on unlabelled normal data. In Stage 2, the fusion module and gating network are jointly optimised to maximise the conditional log-likelihood of normal observations. No fault labels are required at any stage, as illustrated in Figure 5.

### 4.2. CSD Pair-Token Transformer

Given the 16×16 CSD matrix $\hat{M}\left(k\right)$ constructed in Section 3.2, the Coupling Branch extracts a fixed-dimensional coupling feature via physics-aware tokenisation followed by Transformer-based representation learning.

Because $\hat{M}\left(k\right)\in C^{16\times 16}$ is Hermitian, its 16×17/2=136 unique upper-triangular entries contain all the inter-channel coupling information. Each entry is mapped to a four-dimensional real-valued pair token:

(11) tij=Re(M^ij),Im(M^ij),|M^ij|,∠M^ij∈R4,i≤j,

The pair token is then augmented with a physics-aware positional encoding ${\mathrm{PE}}_{ij}=E_{\mathrm{row}}\left(i\right)⊕E_{\mathrm{col}}\left(j\right)⊕E_{\mathrm{block}}\left(\tau \left(i,j\right)\right)$ that concatenates learnable row, column, and block-type embeddings. The block-type function τ(i,j)∈{0,1,2,3} assigns each pair to one of the four physical coupling categories defined in Section 3.2 , so that the attention mechanism can treat different types of physical coupling differently.

The 136 pair tokens are projected into a *d*-dimensional space and fed into a standard Transformer encoder [41]. A learnable classification token $t_{\mathrm{CLS}}$ is added at the beginning to collect global information:

(12) $$H^{\left(0\right)}=\left[t_{\mathrm{CLS}};\;\;W_{t}\;t_{00}+W_{p}\;{\mathrm{PE}}_{00};\;\;\ldots \;;\;\;W_{t}\;t_{15,15}+W_{p}\;{\mathrm{PE}}_{15,15}\right]\;\in \;R^{137\times d},$$

where $W_{t}\in R^{d\times 4}$ and $W_{p}\in R^{d\times 3d_{e}}$ are learnable projection matrices. The encoder consists of $L_{T}$ layers, each comprising multi-head self-attention (MHSA) and a position-wise feed-forward network (FFN) with pre-layer normalisation:

(13) $$\begin{array}{cc} {\hat{H}}^{\left(\ell \right)}\; & =H^{\left(\ell -1\right)}+\mathrm{MHSA}\;\left(\mathrm{LN}(H^{\left(\ell -1\right)})\right), \end{array}$$

(14) $$\begin{array}{cc} H^{\left(\ell \right)}\; & ={\hat{H}}^{\left(\ell \right)}+\mathrm{FFN}\;\left(\mathrm{LN}({\hat{H}}^{\left(\ell \right)})\right), \end{array}$$

where LN(·) denotes layer normalisation and $\ell =1,\ldots ,L_{T}$. The coupling feature vector comes from the classification token at the final layer, and then goes through a two-layer MLP projection head:

(15) $$Z_{\mathrm{CSD}}={\mathrm{MLP}}_{\mathrm{proj}}\;(H_{\left[\mathrm{CLS}\right]}^{\left(L_{T}\right)})\;\in \;R^{d}.$$

The MHSA mechanism has a global receptive field over all 136 pair tokens, and models the dependencies between blocks through the learned attention weights. By examining these weights, we can directly find which coupling paths are most important for anomaly detection.

### 4.3. The Context Branch

The Context Branch treats the pressure signal as a context encoder for the current operating regime. A temporal VQ-VAE performs per-time-step quantisation, which preserves within-window regime transitions that would otherwise be lost under global pooling.

#### 4.3.1. Temporal VQ-VAE

A 1-D convolutional encoder $f_{\phi }$ maps the bivariate pressure signal $P\left(t\right)\in R^{T\times 2}$ to a latent sequence $Z_{e}=f_{\phi }\left(P\right)\in R^{T^{\prime }\times d}$, where $T^{\prime }=16$ for T=1024. Each latent frame is independently quantised against a learnable codebook $C=\{e_{1},\ldots ,e_{K}\}$ with K=8:

(16) $$z_{q}\left(t^{\prime }\right)=e_{k^{*}\left(t^{\prime }\right)},\;k^{*}\left(t^{\prime }\right)=\arg\min_{k}{∥z_{e}\left(t^{\prime }\right)-e_{k}∥}_{2},\;t^{\prime }=1,\ldots ,T^{\prime },$$

The quantisation step gives a codebook index sequence that records the operating-state trajectory, such as idle → biting → steady → tailing. The non-differentiable quantisation step is handled using the straight-through estimator [16]. A symmetric decoder $g_{\psi }$ reconstructs the pressure signal $\hat{P}=g_{\psi }\left(Z_{q}\right)\in R^{T\times 2}$, so that the codebook retains sufficient information for regime representation.

#### 4.3.2. Context Encoder

The quantised sequence $Z_{q}$ is compressed via five families of temporal statistics, namely mean, standard deviation, maximum, minimum, and mean absolute difference:

(17) $$s_{\mathrm{pool}}=\left[{\bar{z}}_{q},\;\;\sigma_{z_{q}},\;\;\max_{t^{\prime }}z_{q}\left(t^{\prime }\right),\;\;\min_{t^{\prime }}z_{q}\left(t^{\prime }\right),\;\;\frac{1}{T^{\prime }\;-\;1}\sum_{t^{\prime }}\;\left|\Delta z_{q}\left(t^{\prime }\right)\right|\right]\in R^{5d}.$$

These statistics are concatenated with raw per-channel pressure statistics $\left[\mu_{P_{1}},\sigma_{P_{1}},\mu_{P_{2}},\sigma_{P_{2}}\right]$ and projected through a two-layer MLP (StatFusion) to produce the context vector:

(18) $$Q_{\mathrm{context}}=\mathrm{StatFusion}\;\left([s_{\mathrm{pool}};\;\mu_{P_{1}},\;\sigma_{P_{1}},\;\mu_{P_{2}},\;\sigma_{P_{2}}]\right)\in R^{d}.$$

The resulting $Q_{\mathrm{context}}$ encodes both the operating-state type through discrete codebook indices and the load magnitude through raw pressure statistics, which gives the complete physical context for the following fusion step.

### 4.4. Physics-Gated Fusion

The consistency and synergy losses are combined through a “physics-gated dynamic weighting scheme” inspired by the Mixture-of-Experts (MoE) paradigm [42]. A lightweight gating network $g_{\theta }$ adjusts the monitoring sensitivity according to the current operating condition. The fusion module consists of three components: a context-conditioned attention layer, a bilateral synergy module, and a gated weighting mechanism.

#### 4.4.1. Context-Conditioned Attention

This module implements the conditional probability model $p_{\theta }\left(Z_{\mathrm{CSD}}|Q_{\mathrm{context}}\right)$ by projecting the context vector as a query that gets the expected coupling state from a memory consisting of both the observed features and a set of learnable reference embeddings.

Specifically, the context vector $Q_{\mathrm{context}}$ is projected as the query, while the coupling feature $Z_{\mathrm{CSD}}$ is augmented with $N_{\mathrm{ref}}$ learnable reference state embeddings $R=\left\{r_{1},\ldots ,r_{N_{\mathrm{ref}}}\right\}\subset R^{d}$ to form the key and value:

(19) $$Z_{\mathrm{expected}}=\mathrm{MHCA}\;(\underset{Q}{\underbrace{Q_{\mathrm{context}}}},\;\;\underset{K,\;V}{\underbrace{\left[Z_{\mathrm{CSD}};\;\;R\right]}})\;\in \;R^{d},$$

where MHCA denotes multi-head cross-attention. The reference embeddings R are initialised from the VQ-VAE codebook centroids and fine-tuned during Stage 2 training. They serve as prototypical coupling states corresponding to the *K* operating-condition types, adding reference embeddings to the key–value memory, which helps stabilise the attention output even if the observed $Z_{\mathrm{CSD}}$ deviates a lot from any normal prototype.

The consistency deviation between the expected and observed coupling features is then computed as:

(20) $$D_{\mathrm{consistency}}={∥Z_{\mathrm{expected}}-Z_{\mathrm{CSD}}∥}_{2}^{2}.$$

Under normal conditions, the context query should retrieve a coupling pattern that closely matches the actual observation, giving a small $D_{\mathrm{consistency}}$. When an anomaly occurs, the observed $Z_{\mathrm{CSD}}$ diverges from the context-predicted pattern, producing a large deviation.

The attention mechanism has two different working modes depending on the operating context. When $Q_{\mathrm{context}}$ indicates a high-pressure steady state, the attention weights concentrate on the reference embedding corresponding to tight coupling, and any deviation of $Z_{\mathrm{CSD}}$ from this compact pattern produces a high consistency score. When $Q_{\mathrm{context}}$ indicates a biting transient, the attention redistributes towards the reference embedding associated with broadband high-energy coupling, and thus can accept the temporary orthogonality relaxation that is a normal physical consequence of the impact event.

#### 4.4.2. Anomaly Scoring and Gated Loss Weighting

In addition to the consistency deviation $D_{\mathrm{consistency}}$, the architecture monitors bilateral coordination between the two guide arms by comparing their respective CSD sub-blocks:

(21) $$D_{\mathrm{synergy}}={∥f_{\mathrm{arm}1}\;\left({\hat{M}}_{\mathrm{Arm}1}\right)-f_{\mathrm{arm}2}\;\left({\hat{M}}_{\mathrm{Arm}2}\right)∥}_{2},$$

where ${\hat{M}}_{\mathrm{Arm}1}$ and ${\hat{M}}_{\mathrm{Arm}2}$ are the 8×8 diagonal sub-blocks corresponding to the channel groups of each arm, and $f_{\mathrm{arm}1},f_{\mathrm{arm}2}:R^{8\times 8}\to R^{d_{s}}$ are learnable MLP projections.

A lightweight gating network $g_{\theta }$ maps the context vector to a pair of non-negative weights:

(22) $$w\left(Q_{\mathrm{context}}\right)=\sigma \;\left(g_{\theta }\left(Q_{\mathrm{context}}\right)\right)⊙w_{\mathrm{base}}+w_{\min},$$

where $g_{\theta }:R^{d}\to R^{2}$ is a two-layer MLP, σ(·) denotes the sigmoid activation, $w_{\mathrm{base}}\in R_{>0}^{2}$ is a learnable base-weight vector, and $w_{\min}>0$ is a constant floor. The anomaly score for a test observation is:

(23) $$S\left(X^{*}\right)=w_{1}\left(Q_{\mathrm{context}}\right)\cdot D_{\mathrm{consistency}}+w_{2}\left(Q_{\mathrm{context}}\right)\cdot D_{\mathrm{synergy}}.$$

The gating mechanism adapts anomaly sensitivity to the operating regime by switching the dominant monitoring channel: during steady-state rolling $w_{1}$ for consistency is increased while $w_{2}$ for synergy is decreased, and during idle periods the pattern reverses, so that at least one monitoring channel always keeps a high weight, forming a complementary strategy. The experimental validation of this regime-aware modulation is presented in Section 5.6.

### 4.5. Training Strategy and Inference

The system is trained in a two-stage self-supervised pipeline summarised in Algorithm 1. Stage 1 pre-trains the two branches independently; Stage 2 jointly optimises the fusion module, gating network, and StatFusion layer.
**Algorithm 1** Two-Stage Self-Supervised Training Pipeline  **Input:** Normal training set $D={\left\{X_{i}\right\}}_{i=1}^{N}$; hyperparameters *m*, β, $\lambda_{r}$, $\lambda_{d}$, $E_{w}$  *% — Stage 1a: CSD Transformer Pre-training —* 1: **for** epoch =1
**to**
$E_{1a}$ **do**
 2:  Construct positive pairs from temporally adjacent CSD matrices $\left({\hat{M}}_{t},{\hat{M}}_{t+1}\right)$
 3:  Generate hard negatives by injecting orthogonality noise into *P*–*V* coupling blocks
 4:  Update CSD Transformer via $L_{\mathrm{triplet}}=\max(0,\;∥z_{a}-z_{p}∥-∥z_{a}-z_{n}∥+m)$
 5: **end for**  *% — Stage 1b: Temporal VQ-VAE Pre-training —* 6: **for** epoch =1
**to**
$E_{1b}$
**do**▹ Early stopping, patience = 15 7:  Update VQ-VAE via $L_{\mathrm{VQ-VAE}}=L_{\mathrm{VQ}}+\beta \;L_{\mathrm{commit}}+\lambda_{r}\;L_{\mathrm{recon}}+\lambda_{d}\;L_{\mathrm{div}}$ 8: **end for**▹ StatFusion receives no gradient in Stage 1  *% — Stage 2: Conditional Joint Fine-tuning —* 9: Load pre-trained CSD Transformer and VQ-VAE weights; randomly initialise fusion, gating, StatFusion10: **for** epoch =1
**to**
$E_{2}$ **do**11:  **if** epoch $\leq E_{w}$
**then**▹ Gating warm-up12:    $w\leftarrow w_{\mathrm{base}}+w_{\min}$▹ Fixed weights13:  **else**14:    w← gating network output (Equation (22))15:  **end if**16:  $L_{\mathrm{total}}=w_{1}\cdot {∥Z_{\mathrm{expected}}-Z_{\mathrm{CSD}}∥}_{2}^{2}+w_{2}\cdot D_{\mathrm{synergy}}$17:  Update all parameters end-to-end▹ Gradients flow to StatFusion via MHCA18: **end for**   **Output:** Trained model; discard VQ-VAE decoder for inference

The joint fine-tuning loss (Stage 2, line 15) combines the consistency and synergy objectives with context-dependent weights:

(24) $$L_{\mathrm{total}}=w_{1}\left(Q_{\mathrm{context}}\right)\cdot {∥Z_{\mathrm{expected}}-Z_{\mathrm{CSD}}∥}_{2}^{2}+w_{2}\left(Q_{\mathrm{context}}\right)\cdot D_{\mathrm{synergy}}.$$

The triplet loss in Stage 1a is preferred over InfoNCE [10] because the limited diversity of normal-condition CSD matrices can cause InfoNCE to converge to a trivially uniform distribution. The VQ-VAE loss in Stage 1b combines codebook alignment ($L_{\mathrm{VQ}}$), encoder commitment ($L_{\mathrm{commit}}$), pressure reconstruction ($L_{\mathrm{recon}}$), and a KL-based codebook diversity penalty $L_{\mathrm{div}}=\mathrm{KL}\left(P_{\mathrm{usage}}∥U\left(K\right)\right)$ to prevent mode collapse. One important design point is that the StatFusion layer remains dormant during Stage 1 and receives its first meaningful gradients only in Stage 2 via the chain $L_{\mathrm{total}}\to Z_{\mathrm{expected}}\to \mathrm{MHCA}\to Q_{\mathrm{context}}\to \mathrm{StatFusion}$. The gating warm-up (lines 11–14) stops the randomly initialised gating network from causing instability in early joint training. At inference, the VQ-VAE decoder is discarded and each window is scored in a single feed-forward pass via Equation (23); an observation is flagged as anomalous when $S(X^{*})$ exceeds a threshold ρ set using Peaks-Over-Threshold extreme-value analysis.

## 5. Experimental Evaluation

### 5.1. Experimental Setup

#### 5.1.1. Dataset Description

**Figure 6:**
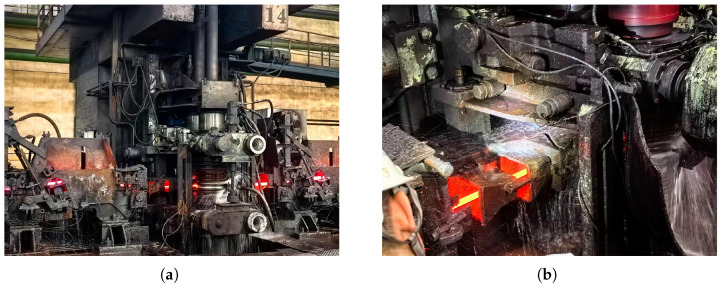
Data collection environment on the operational hot rolling line. (**a**) Overview of a rolling stand on the hot rolling production line. (**b**) Intelligent roller guide during active rolling.

All experiments are conducted on data collected from the intelligent roller guide system described in Section 3, deployed on an operational hot-rolling production line. The four-channel sensor array ($P_{1}$, $V_{1}$, $P_{2}$, $V_{2}$) acquires data at a sampling rate of $f_{s}=100$ Hz, and each observation window spans T=1024 samples, approximately 10.24 s. During training, windows are extracted with 87.5% overlap (stride = 128), yielding a total of 173,737 training samples of normal operating data. At inference time, a more dense step size (10 samples, i.e., 0.1 s) is adopted to enhance the temporal resolution. This dataset covers all typical operating conditions encountered in the production process, including idle periods, low-pressure calibration runs, high-pressure steady-state rolling, bite transient events, and tail events. Figure 6 shows the actual data acquisition environment, confirming that this dataset comes from an in-service industrial production line rather than a controlled laboratory environment.

In addition, a separate test set was constructed. This test set was achieved by retaining 20% of the normal data, i.e., 8687 windows, and supplementing it with 900 synthetic fault windows at 300 per fault type, generated according to the methodology described in Section 5.1.2. Among them, the validation set contains 10% of the normal data, i.e., 4344 windows, for threshold calibration and model selection.

As shown in Figure 4 (Section 3), the original four-channel signals present characteristic non-stationary patterns caused by repetitive rolling processes. The significant amplitude changes and obvious transient events highlight the challenges in distinguishing genuine mechanical faults from benign operational state changes. To quantify this industrial non-stationarity, Table 2 summarizes the statistical characteristics of the multimodal signals under distinct rolling regimes. The substantial increases in pressure variance and vibration RMS during the biting transient, relative to idle operation, motivate context-conditioned modeling rather than simple fixed-threshold methods.

#### 5.1.2. Synthetic Fault Injection Methodology

A major difficulty in evaluating anomaly detection systems for industrial equipment is the scarcity of real-world fault data. Well-maintained industrial assets exhibit a “high-reliability paradox”: the higher the maintenance standard, the fewer fault samples are available for training and evaluating diagnostic models [5,9]. During the monitoring period of the present study, the deployed sensor system captured only a small number of fault episodes, which was insufficient to form a statistically meaningful test set.

To address this limitation, we adopted an evidence-based synthetic fault construction protocol rather than generating faults arbitrarily. Specifically, we consulted experienced on-site maintenance operators and combined their reports with signal-level analysis of the limited available fault recordings. This process identified three representative fault types and their characteristic signal signatures, from which we designed channel-specific amplitude transforms for the four-channel sensor signals.

This synthetic fault injection method based on expert knowledge follows common practice in machinery condition monitoring. The widely adopted CWRU bearing benchmark [43] relies on artificially seeded defects to evaluate diagnostic algorithms in the absence of naturally occurring run-to-failure data. More recently, Wang et al. [44] showed that combining domain expertise and domain adaptation on real operational vibration data can produce physically realistic training samples, and Ali et al. [45] proposed signature-guided data augmentation for motor diagnostics, further supporting physics-informed synthetic fault generation for industrial applications. Recent studies also employ simulation-to-reality transfer and digital twin methodologies to mitigate the scarcity of measured fault data [46,47].

Each synthetic fault is generated by selecting a 30-s segment, i.e., 3000 samples, from the normal dataset and applying channel-specific amplitude transforms that replicate the characteristic signal signatures distilled from on-site fault observations and operator knowledge. Experienced on-site operators verified that the injected signatures were consistent with the limited field fault recordings. Table 3 summarizes this comparison. The power spectral density (PSD) cosine similarity exceeds 0.86 for all fault types, and the spectral kurtosis values are closely matched between synthetic and recorded faults. These results indicate that the synthetic transformations preserve the main frequency-domain and impulsive characteristics of the observed mechanical degradation. Table 4 summarises the three fault types, their physical meanings, and the corresponding mathematical transforms.

**Figure 7:**
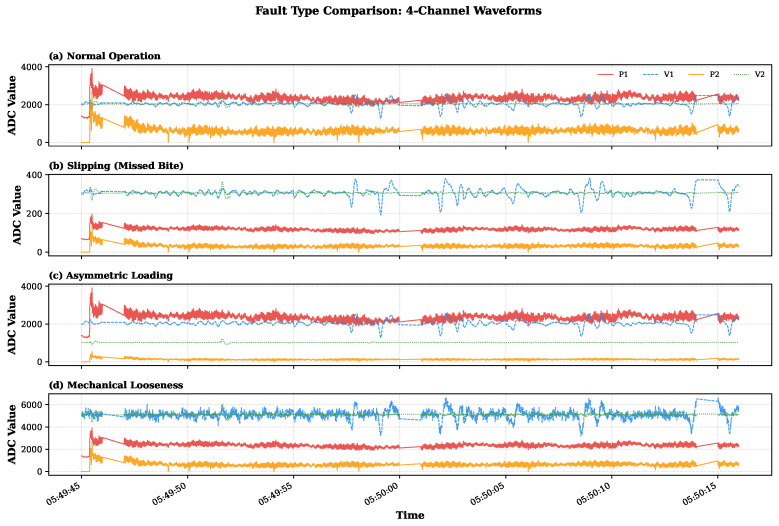
Comparison of four-channel waveforms under normal operation and three synthetic fault conditions. The panels compare normal operation with Slipping, Asymmetric Loading, and Mechanical Looseness, and the sensor-channel colors correspond to $P_{1}$, $V_{1}$, $P_{2}$, and $V_{2}$ as shown in the legend in panel (**a**).

In the proposed architecture, the three fault types activate different diagnostic pathways. Slipping causes bilateral signal collapse and appears in the CSD structure as an almost complete loss of inter-channel coupling, which is primarily detected by the coupling branch. Asymmetric Loading breaks the bilateral symmetry while preserving the overall signal energy, and is therefore detected mainly through the synergy module ($D_{\mathrm{synergy}}$ in Equation (21)). Mechanical Looseness injects broadband vibration energy without altering the pressure channel, creating a cross-modal feature that single-modal methods cannot detect but that remains visible in the coupling analysis. Figure 7 shows the side-by-side comparison of all three types of normal and fault injection waveforms, confirming that each synthetic fault produces physically consistent signal changes.

### 5.2. Implementation Details

The entire system is implemented in PyTorch 2.8.0 with CUDA 12.8 on a workstation equipped with an NVIDIA RTX 5880 Ada Generation GPU (48 GB memory). The CSD Transformer uses d=64, 4 attention heads, and $L_{T}\;=\;3$ encoder layers over 136 four-dimensional pair tokens, with a triplet margin of 0.5 and perturbation strength of 0.5. The temporal VQ-VAE has a codebook of K=8 entries, temporal resolution $T^{\prime }\;=\;16$, and a Conv1d encoder with channel progression 2→32→64→128 and stride 4. The gating network is a two-layer MLP of dimensions 128→32→2 with weight floor $w_{\min}\;=\;0.1$ and base weights [1.0,0.5]. All stages use the AdamW optimiser with a batch size of 64; the learning rates are $5\;\times \;10^{-4}$ for Stage 1a, $10^{-3}$ for Stage 1b, and $10^{-4}$ for Stage 2 with cosine annealing and warm restarts.

Table 5 lists the hyperparameters used in signal preprocessing, network architecture, and optimization, including the db4 wavelet basis and the VQ-VAE loss coefficients.

Training follows the two-stage pipeline in Algorithm 1: Stage 1a pre-trains the CSD Transformer for 30 epochs with triplet loss, Stage 1b pre-trains the VQ-VAE for up to 50 epochs with early stopping patience of 15, and Stage 2 jointly fine-tunes the full model for 100 epochs using the context-gated loss (Equation (24)) with a cosine annealing scheduler. The best model is selected at epoch 68. The total training time is approximately 3.6 h on a single NVIDIA RTX 5880 Ada GPU (48 GB). The per-window end-to-end processing time is approximately 8 ms, including CSD matrix construction and model inference. At inference time, a dense sliding window with stride = 10, i.e., 0.1 s, is employed, yielding 10 anomaly-score updates per second with a total compute load of 80 ms s^−1^, corresponding to 8% GPU utilisation.

The dense inference stride produces a new anomaly score every $\Delta t=\mathrm{stride}/f_{s}+t_{\mathrm{proc}}=10/100+0.008\approx 108$ ms. This 108 ms response cycle represents a 12× improvement over the training-time stride of 128 and is within the sub-second response requirements of real-time industrial rolling mill monitoring.

### 5.3. Comparison with Baseline Methods

In order to show that the proposed method is effective, we compare it against eleven representative unsupervised or self-supervised anomaly detection methods covering two categories: one traditional signal-processing baseline and ten deep learning methods. FFT + Fixed Threshold applies per-channel spectral energy thresholding. The deep learning baselines include DAGMM [48], USAD [49], OmniAnomaly [50], Anomaly Transformer [13], TranAD [51], DCdetector [31], TimesNet [32], DACR [33], KAN-AD [52], and CAROTS [30].

All baseline methods are trained on the same normal training set and evaluated on the same test set containing both normal samples and the three synthetic fault types described in Section 5.1.2. For each deep learning baseline, we use the original authors’ recommended hyperparameters and tune only the anomaly threshold to optimise the F1 score on a held-out validation set.

All baseline methods receive the raw four-channel time-domain signals ($P_{1}$, $V_{1}$, $P_{2}$, $V_{2}$) as input, as this is the standard configuration described in their respective publications. The proposed method additionally employs wavelet packet decomposition-based channel expansion and CSD matrix construction as a physics-informed preprocessing pipeline. To ensure a fair and comprehensive comparison, we evaluate all baseline methods within the same complete pipeline by providing them with the expanded 16-channel CSD representation instead of the raw 4-channel time-domain signals. Additionally, we introduce Magnitude-Squared Coherence (MSC) as a classical frequency-domain baseline. To guarantee statistical reliability, all deep learning models are independently trained using five different random seeds, and the results are reported as mean ± standard deviation. A paired *t*-test on the AUC-ROC scores confirmed that our proposed method is statistically significantly better than the strongest baseline CAROTS (p<0.01).

Table 6 presents the updated results.

The proposed method achieves the highest score in all four evaluation metrics, especially in the false-alarm metric that is most critical for industrial deployment. Compared with the strongest baseline CAROTS [30], our method improves AUC-ROC from 0.941 to 0.952, AUC-PR from 0.912 to 0.921, and F1 from 0.910 to 0.912, while reducing FPR@95%TPR from 0.062 to 0.048, corresponding to a 22.6% relative reduction in false alarms at the same 95% TPR operating point.

Among the deep learning baselines, USAD reaches an AUC-ROC of 0.901 with a relatively high FPR of 0.115, indicating that its adversarial reconstruction remains sensitive to benign regime transitions. OmniAnomaly improves the multimodal modelling of normal states, achieving an AUC-ROC of 0.906 and FPR of 0.095. Among the Transformer-style baselines, Anomaly Transformer achieves a slightly higher AUC-PR than TimesNet (0.901 vs. 0.898), whereas TimesNet gives the higher F1 score (0.902 vs. 0.899), which suggests that stronger ranking quality does not necessarily translate into the best thresholded classification behaviour. DCdetector attains the highest AUC-ROC and AUC-PR among the pre-2025 Transformer baselines at 0.930 and 0.905, while TranAD yields a marginally higher F1 of 0.905 at a slightly lower AUC-ROC of 0.929. KAN-AD further reduces FPR@95%TPR to 0.070 and improves AUC-PR to 0.907, but still remains below CAROTS overall.

CAROTS remains the strongest baseline overall, with an AUC-ROC of 0.941 and FPR@95%TPR of 0.062, yet it still cannot match the proposed method at the low-false-alarm operating point. A likely reason is that CAROTS learns causal invariance mainly through augmentation-based contrastive objectives, whereas it does not explicitly condition the expected coupling pattern on the current load regime. Consequently, benign load transitions can still move its anomaly score toward the fault boundary, while the proposed physics-gated model adjusts the consistency and synergy sensitivities according to the pressure-derived context. This shows that implicit causal modelling alone is insufficient to replace explicit physics-gated conditioning for suppressing false alarms under strongly non-stationary industrial conditions.

### 5.4. Ablation Study

We conduct two groups of ablation experiments to validate the architectural design and the training strategy, respectively.

#### 5.4.1. Architecture Ablation: Necessity of the Dual-Branch Design

To prove that both the coupling branch and the context branch are indispensable, we evaluated six configurations: the complete model (A1), the configuration containing only the coupling branch without context information (A2), the configuration containing only the context branch without CSD features (A3), a variant using the original 4×4 CSD matrix without virtual channel expansion (A4), a variant without the synergy term (A5), and a variant using a static fixed fusion strategy instead of the physics-gated network (A6). The results are listed in Table 7.

Five key findings emerge from the architecture ablation:

1. Both branches are indispensable. If the coupling branch, variant A2, or the context branch, variant A3, is deleted, the performance will drop significantly: AUC-ROC decreases by 6.1% and 7.9% respectively, while the false alarm rate increases by 2.3× and 2.8× compared to the complete model. The coupling-only variant A2 still maintains a certain anomaly recognition ability, but during the transition of operating conditions, it will have a higher false alarm rate due to the inability to distinguish normal instantaneous coupling changes from fault-induced anomalies. On the contrary, the context-only variant A3 has the worst AUC-ROC situation, as relying solely on pressure domain statistics cannot detect mechanical degradation mainly manifested in the coupling structure of the vibration domain.
2. The dual-branch fusion approach brings synergy enhancement. The AUC-ROC value of the entire model, 0.952, is significantly higher than the better of the two single-branch variants, A2 at 0.891, indicating that the physical gating fusion module extracts complementary information from the two branches rather than merely averaging their outputs.
3. Virtual channel expansion is beneficial. Degrading the CSD matrix from the expanded 16×16 to the original 4×4 in variant A4 reduces AUC-ROC by 3.4% and increases FPR by 58%. The wavelet packet decomposition-based channel expansion makes the frequency-domain representation richer, giving the Transformer more detailed sub-band coupling patterns that help improve fault discrimination.
4. The synergy term is essential for detecting asymmetric anomalies. Variant A5, which removes the synergy term, experiences a significant drop in AUC-ROC to 0.902 and an increase in FPR to 0.104. This is because under asymmetric loading conditions, one side experiences a load drop, causing the context branch to incorrectly predict an “idle” state. The corresponding drop in vibration matches this idle expectation, masking the fault in the consistency evaluation. The synergy term resolves this by directly comparing the physical symmetry between the two arms, effectively preventing such missed detections.
5. Dynamic physics-gated fusion outperforms static weights. Variant A6, which uses fixed weights ($w_{1},w_{2}$) instead of the context-driven gating network, achieves an AUC-ROC of only 0.915. The dynamic gating mechanism allows the model to non-linearly amplify the most reliable monitoring branch according to the current operating regime (e.g., amplifying consistency during steady rolling and synergy during idle periods), avoiding the feature dilution inherent in static weighting.

#### 5.4.2. Pre-Training Strategy Ablation

To validate the two-stage pre-training strategy, we compare the full training pipeline with variants that skip one or both pre-training stages. Table 8 presents the results.

The complete two-stage pre-training pipeline gives a total improvement of +5.5% in AUC-ROC and a 55% reduction in FPR relative to training from scratch, variant C2. Without any pre-training, all modules are randomly initialised and must learn modality-specific representations and cross-modal fusion simultaneously, which causes convergence difficulties and not ideal feature quality. The FPR increases to 0.107, more than 2.2× that of the full model.

Skipping VQ-VAE pre-training, variant C3, results in a 2.1% AUC-ROC drop and a 31% FPR increase from 0.048 to 0.063. Without a properly initialised codebook, the VQ-VAE has difficulty finding meaningful operating-condition prototypes during joint fine-tuning, degrading the quality of the context vector $Q_{\mathrm{context}}$ and consequently the precision of the gating mechanism. Skipping CSD pre-training, variant C4, has an even larger impact on AUC-ROC (−2.8%), which shows that the contrastive pre-training is necessary for the Transformer to learn discriminative coupling representations before the more complex joint optimisation.

These results support the sequential, modular training strategy: each branch needs to first build high-quality, modality-specific representations, and only then can the fusion module well learn the conditional relationship between operating context and expected coupling state.

### 5.5. Sensitivity Analysis

**Figure 8:**
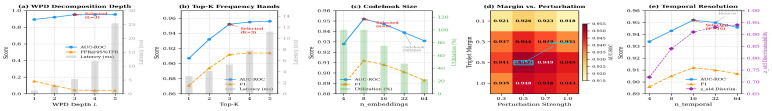
Sensitivity analysis of five key hyperparameters arranged in a single row. From left to right, the panels show the decomposition depth *L*, Top-*K* sub-band selection, VQ-VAE codebook size, triplet loss setting, and temporal resolution. Different colors denote the evaluation metrics shown in the legends, including the F1 score in panel (c), and selected configurations are marked with ★.

We investigate the sensitivity of the proposed system to five critical hyperparameters: the Top-*K* sub-band selection factor, the wavelet packet decomposition depth *L*, the VQ-VAE codebook size, the triplet loss margin and perturbation strength, and the VQ-VAE temporal resolution. For each parameter, all other settings are held at their default values specified above. Figure 8 presents the decomposition-depth analysis together with the remaining four sensitivity analyses.

Figure 8 first shows that increasing the decomposition depth from L=1 to L=3 improves the AUC-ROC and substantially reduces FPR@95%TPR, indicating that three-level wavelet packet decomposition provides sufficient high-frequency sub-band resolution for isolating fault-related resonance components. Further increasing *L* to 4 or 5 brings negligible performance gains, while the preprocessing time rises rapidly due to the exponential growth of $2^{L}$ sub-bands. Therefore, L=3 strikes the optimal balance between discriminative frequency resolution and real-time computational efficiency. The remaining panels in Figure 8 present the sensitivity analysis across the other four key hyperparameters.

For the top-*K* sub-band selection (the second panel in Figure 8), increasing *K* from 1 to 3 gives obvious AUC-ROC improvements, from 0.907 to 0.952, by introducing finer frequency-domain coupling information. Beyond K=3, the improvement is very small, with only +0.3% for K=5, while the per-window processing time increases from approximately 8 to 13 ms. K=3, i.e., 16 virtual channels, has the best balance between performance and efficiency.

For the VQ-VAE codebook size (the third panel in Figure 8), performance peaks at K=8 with 100% codebook utilisation. Smaller codebooks with K=4 mix up distinct operating states, while larger ones with K≥ 16 suffer from progressive codebook collapse, as utilisation drops from 75% to 23%, indicating that K=8 naturally matches the number of physically distinct operating regimes. This naturally aligns with the number of physically distinct operating regimes observed in the rolling process, such as idle, biting transient, steady-state rolling under various load magnitudes, and tailing.

For the triplet margin and perturbation strength (the fourth panel in Figure 8), the two parameters jointly control the difficulty of contrastive learning. Weak perturbation at ϵ=0.3 gives negatives that are not different enough, while excessive values m=1.0, ϵ=1.0 cause overfitting to the specific perturbation distribution. The selected configuration m=0.5, ϵ=0.5 balances discriminative power with generalisation.

For the VQ-VAE temporal resolution (the fifth panel in Figure 8), low resolution at $T^{\prime }\;=\;4$, corresponding to ∼2.5 s per frame, averages out transient events, giving poor state discriminability with $z_{\mathrm{std}}\;=\;0.72$. Beyond $T^{\prime }\;=\;16$, too fine resolution captures irrelevant intra-state fluctuations. $T^{\prime }\;=\;16$, corresponding to ∼0.64 s per frame, matches the typical duration of rolling-process transitions. A temporal resolution of $T^{\prime }\;=\;16$ optimally captures the typical transition timescales between distinct rolling states, avoiding both the over-smoothing of transients at lower resolutions and the excessive noise sensitivity at higher ones.

### 5.6. Robustness and Interpretability Analysis

Beyond anomaly-detection accuracy, practical deployment also depends on whether the embedded sensors remain stable under prolonged thermal and vibrational loading. Table 9 summarizes the in-situ durability metrics collected over the monitoring campaign. The observed changes in linearity, bias, and vibration SNR remained limited, and no sensor failures occurred. These results support the feasibility of minimally invasive embedding with hermetic stainless-steel protection in the hot-rolling environment. Because no sensor failures were observed in service, fault tolerance was not evaluated empirically; nevertheless, the model can still exploit bilateral pressure asymmetry through the synergy branch ($D_{\mathrm{synergy}}$) if one vibration channel becomes unavailable.

**Figure 9:**
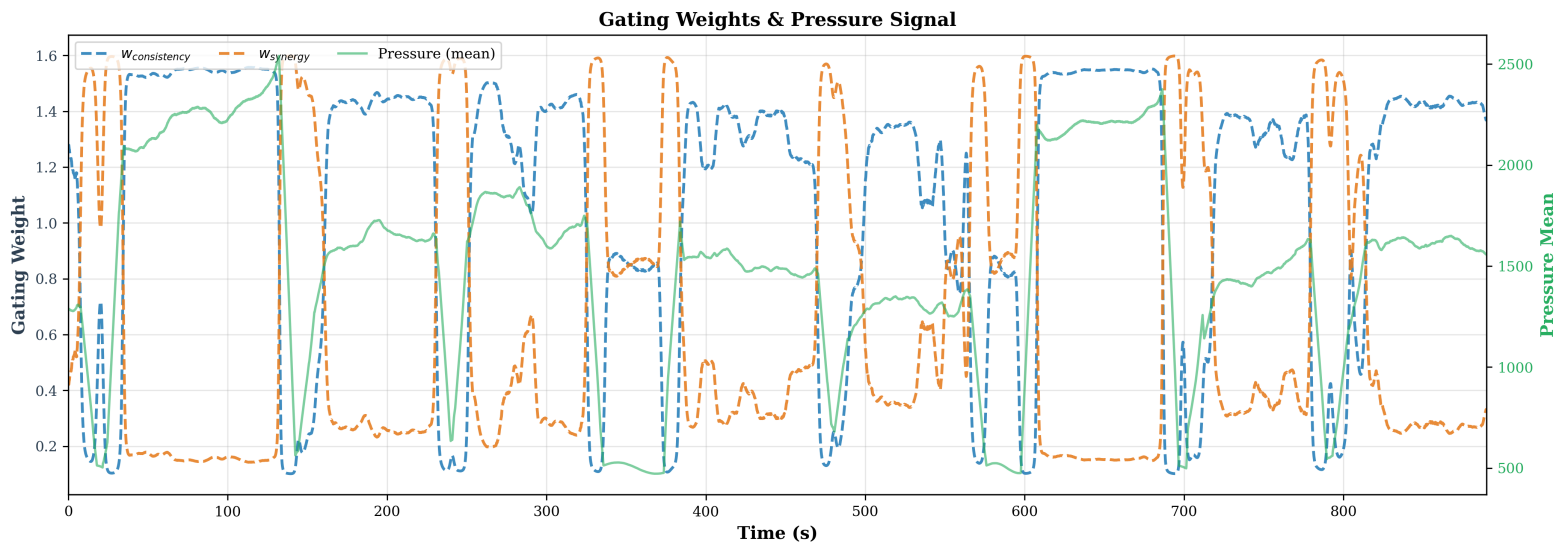
Temporal co-evolution of the gating weights $w_{\mathrm{consistency}}$ and $w_{\mathrm{synergy}}$ with the mean pressure signal over ∼900 s of continuous operation. The blue dashed curve denotes $w_{\mathrm{consistency}}$, the orange dashed curve denotes $w_{\mathrm{synergy}}$, and the green solid curve denotes the mean pressure signal.

Figure 9 overlays the two learned gating weights, $w_{\mathrm{consistency}}$ and $w_{\mathrm{synergy}}$, on top of the mean pressure signal across ∼900 s of continuous operation. The two weights show a clear anti-correlated pattern: during idle periods $w_{\mathrm{consistency}}$ stays near its floor, ∼0.1–0.2, while $w_{\mathrm{synergy}}$ peaks around ∼1.5–1.6; during steady-state rolling the pattern reverses, with $w_{\mathrm{consistency}}$ at its maximum around ∼1.4–1.6 and $w_{\mathrm{synergy}}$ suppressed to ∼0.2–0.6. This complementary modulation helps ensure that at least one monitoring channel remains active: coupling-fidelity monitoring dominates when the cross-spectral pattern is most predictable under load, while bilateral symmetry monitoring takes over during no-load intervals when the two arms should be in identical resting states.

This complementary sensitivity modulation is the main reason for the 22.6% FPR reduction relative to CAROTS, from 0.062 to 0.048. Removing the context branch, variant A2 in Table 7, causes FPR to surge to 0.112, a 133% increase, which shows the gating module’s important role in reducing false alarms during non-stationary intervals.

**Figure 10:**
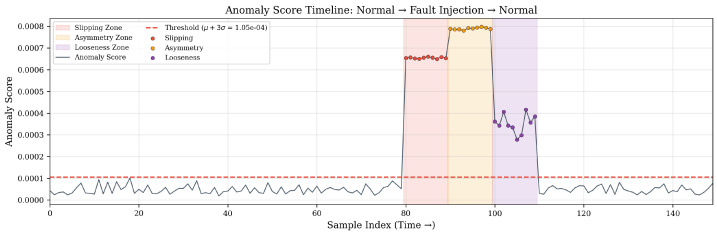
Anomaly score timeline over a test segment containing normal operation and synthetic fault injections. The gray-blue solid line denotes the anomaly score, and the red dashed line denotes the detection threshold. The light red, light orange, and light purple shaded regions indicate the injection intervals for Slipping, Asymmetric Loading, and Mechanical Looseness, respectively; the red, orange, and purple circular markers denote the corresponding fault samples.

Figure 10 overlays the context-gated anomaly score on a test segment containing normal operation and three synthetic fault injections. The shaded regions explicitly denote the Slipping, Asymmetric Loading, and Mechanical Looseness injection intervals, making it possible to visually align each score plateau or spike with the corresponding fault event. The score remains below the threshold during all normal conditions, including biting transients. Among fault types, Asymmetric Loading gives the highest score increase because it disrupts both bilateral symmetry and cross-modal coupling simultaneously; Slipping gives moderate scores as the bilateral collapse preserves partial symmetry; Mechanical Looseness produces the lowest but still detectable response, which is in line with its more subtle vibration-only feature.

**Figure 11:**
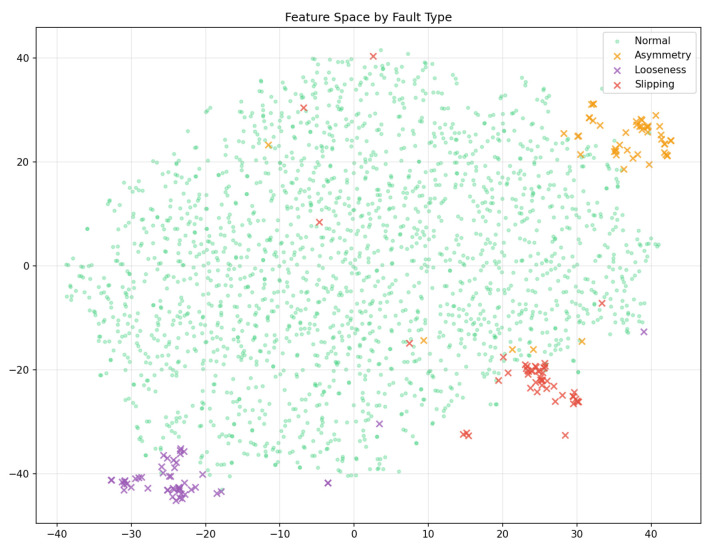
t-SNE visualisation of CSD Transformer features coloured by fault type.

Figure 11 presents a t-SNE projection of the CSD Transformer output features $Z_{\mathrm{CSD}}$. The three fault types form tight, well-separated clusters, visibly distant from the diffuse cloud of normal samples. This separation appears without any fault labels during training, which shows that the self-supervised objective can naturally produce fault-discriminative representations.

**Figure 12:**
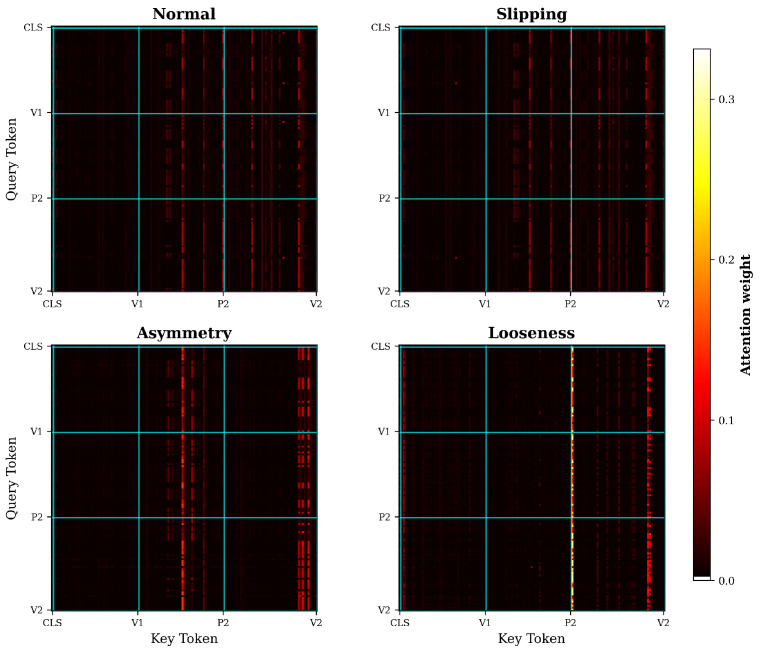
CSD Transformer attention maps under four conditions. The sequence comprises 137 tokens: 1 classification token and 136 sensor-pair tokens. Cyan lines mark physical block boundaries, and the shared colorbar reports the attention weight on an explicit 0.0–0.3 scale.

Figure 12 visualises the CSD Transformer attention maps under four conditions. Under normal operation, attention distributes broadly across multiple sensor-pair groups. Under Slipping, the distribution remains similar overall, with a few isolated tokens exhibiting anomalously high weights due to near-zero CSD magnitudes amplifying softmax sensitivity. Asymmetric Loading concentrates attention on $V_{2}$-related pair tokens corresponding to the affected arm. Mechanical Looseness collapses attention to a single intense band at the $P_{2}$ block boundary, which points to the cross-modal orthogonality violation.

To explicitly reveal the diagnostic difficulty of different fault types, Table 10 presents the detailed confusion matrix for the test set at the calibrated threshold. As shown, Slipping achieves the highest detection rate (288/300), because the simultaneous collapse of both the pressure and vibration channels causes extreme violations in the consistency branch. Asymmetric Loading and Mechanical Looseness show slightly lower, yet robust, detection rates (282/300 and 285/300, respectively). Asymmetric Loading is mainly captured by the synergy module due to unilateral pressure drops, while Mechanical Looseness is detected by the coupling branch as it injects cross-modal broadband vibration without altering the pressure expectation. The false alarm rate on normal data is bounded at 0.048 (417/8687), confirming that the physics-gated architecture successfully discriminates between genuine mechanical degradation and benign non-stationary operating transients.

## 6. Conclusions

### 6.1. Summary

This paper presented a cyber-physical system that combines an embedded orthogonal sensing layout (P⊥V) with a dual-branch self-supervised anomaly detection architecture for roller guide monitoring under label-scarce, non-stationary industrial conditions. By treating pressure as a context condition rather than a directly monitored variable, the framework changes anomaly assessment from unconditional to conditional density estimation. Experimental evaluation on a dataset comprising real operational data and expert-validated synthetic fault scenarios showed an AUC-ROC of 0.952, an F1 of 0.912, and an FPR@95%TPR of 0.048, which improves over the strongest baseline CAROTS by 0.011 in AUC-ROC and reduces the false positive rate at the 95% TPR operating point from 0.062 to 0.048. Ablation studies showed that both branches are necessary, and so is the two-stage pre-training pipeline, yielding +5.5% AUC-ROC, while fault-specific attention patterns showed physically meaningful interpretability.

### 6.2. Robustness and Deployment Challenges

Deployment in hot rolling environments involves substantial environmental and operational variability (EOV), as highlighted by recent domain-adaptive frameworks in structural health monitoring [53]. The in-situ monitoring campaign showed only limited changes in sensor linearity, bias, and vibration SNR, with no sensor failures during service. These observations support the practical feasibility of minimally invasive embedding with hermetic stainless-steel protection under sustained thermal and vibrational loading.

### 6.3. Future Work

Two main limitations of this study point to the following research directions: (i) the quantitative evaluation relies on synthetic fault injection rather than a large corpus of real fault recordings, and (ii) all experiments are confined to a single hot-rolling production line. Environmental and operational variability further complicates representation learning under normal-only training. The proposed physics-gated architecture partly mitigates this effect through context-dependent modulation, consistent with recent work on EOV in structural health monitoring [53].

Based on these limitations, the following directions for future research are suggested:

1. **Real fault data collection and validation.** The most urgent next step is to accumulate a sufficient corpus of real fault recordings through long-term deployment of the monitoring system. As genuine fault events are captured over time, they can be used to gradually verify and adjust the detection thresholds, and eventually replace the synthetic fault evaluation with a fully realistic assessment.
2. **Multi-line and cross-equipment transfer.** Deploying the system on additional production lines with different rolling configurations can test whether the learned representations can transfer. Domain adaptation or few-shot fine-tuning strategies may be explored to accelerate deployment on new lines with minimal data collection effort. A practical strategy is to freeze the base weights of the CSD Transformer while fine-tuning the VQ-VAE codebook and reference embeddings to accommodate different absolute load levels and mechanical baseline states.
3. **Fault identification and severity estimation.** The fault-specific attention patterns observed in the interpretability analysis (Section 5.6) suggest that the learned feature space has enough discriminative information for not only fault detection but also fault type identification. Future work could explore semi-supervised or few-shot classification heads that make use of these representations to give operators useful diagnostic information in addition to binary anomaly alerts.

## Figures and Tables

**Table 1:** Detailed specifications of the selected sensors for the intelligent roller guide system.

Parameter	Pressure: F1005-20000	Vibration: WKD0181
Transducer type	Resistive strain gauge	Piezoelectric (shear-mode ceramic)
Signal output	Full-bridge Wheatstone	Charge (∼35 pC/g)
Measurement range	20 kN	±1000 g
Resonant frequency	—	30 kHz
Nonlinearity	0.5% F.S.	0.5% F.S.
Mounting	Face-fed precision slot	M5 threaded hole
Encapsulation	Hermetic stainless steel	Stainless steel (laser-welded)
Dimensions	Low-profile button type	⌀16 × 22 mm
Operating temp.	Customisable for high temp.	Customisable for high temp.

**Table 2:** Statistical characteristics of multi-modal signals across distinct rolling regimes extracted from the normal training set.

Operating Condition	Pressure Mean	Pressure Variance	Vibration RMS
Idle	533.06	24,093.93	1022.30
Biting Transient	1059.99	847,746.86	3033.37
Steady-State Rolling	2523.75	486,757.35	2042.75
Tailing Transient	1056.58	821,055.31	2961.14

**Table 3:** Quantitative similarity between synthetic fault signatures and real fault recordings.

Fault Type	Evaluation Metric	Synthetic Fault	Real Fault
Slipping	Cosine Similarity of PSD	0.89	
	Spectral Kurtosis	6.45	6.38
Asymmetric Loading	Cosine Similarity of PSD	0.91	
	Spectral Kurtosis	6.42	6.29
Mechanical Looseness	Cosine Similarity of PSD	0.86	
	Spectral Kurtosis	5.70	5.85

**Table 4:** Synthetic fault types derived from on-site operator experience and limited fault recordings, with physical interpretations and mathematical transforms applied to normal signal segments.

Fault Type	Physical Meaning	Transform	Rationale
Slipping	Billet fails to be gripped; contact force drops to near-zero	$P_{1,2}\;\times \;0.05$, $V_{1,2}\;\times \;0.15$	Bilateral pressure and vibration collapse due to loss of excitation
Asymmetric Loading	Misalignment or uneven temperature causes one-sided overload	$P_{2}\;\times \;0.20$, $V_{2}\;\times \;0.50$	One arm load drops while the other remains unchanged
Mechanical Looseness	Bearing wear or bolt loosening causes excess vibration	$V_{1,2}\;\times \;2.5+n$, $n\;∼\;N(0,1.5\sigma_{V})$	Vibration ~2.5× increase with HF noise; pressure unaffected

**Table 5:** Experimental hyperparameters and network dimensions.

Category	Parameter	Value
Signal Preprocessing & WPD	Sampling Rate ($f_{s}$)	100 Hz
	Window Size (*T*)	1024
	Stride	Training: 128, Inference: 10
	Wavelet Basis	db4 (Daubechies 4)
	Decomposition Depth (*L*)	3
	Top-*K* Sub-bands Selection	3
VQ-VAE Branch	Temporal Resolution ($T^{\prime }$)	16
	Codebook Size (*K*)	8
	Embedding Dimension (*d*)	64
	Reconstruction Loss Coeff. ($\lambda_{r}$)	1.0
	Commitment Loss Coeff. (β)	0.25
	Diversity Penalty Coeff. ($\lambda_{d}$)	0.1
CSD Transformer	Sensor Pair Tokens	136
	Transformer Layers ($L_{T}$)	3
	Attention Heads	4
	Triplet Loss Margin (*m*)	0.5
	Perturbation Strength (ϵ)	0.5
Training & Optimization	Optimizer	AdamW
	Batch Size	64
	Learning Rates	$5\times 10^{-4}$ (VQ-VAE), $10^{-3}$ (CSD), $10^{-4}$ (Joint)
	Epochs	30 (VQ-VAE), 50 (CSD), 100 (Joint)
	Early Stopping Patience	15

**Table 6:** Comparison with baseline anomaly detection methods. The best result in each column is highlighted in **bold**. ↓ indicates lower is better. Note: All methods are trained exclusively on normal operating data. All deep learning baselines have been retrained using the unified 16-channel CSD representation to separate the contribution of the network architecture from the preprocessing pipeline.

Category	Method	AUC-ROC	AUC-PR	F1	FPR@95%TPR ↓
Traditional	FFT + Threshold	0.764 ± 0.012	0.723 ± 0.015	0.738 ± 0.014	0.284 ± 0.021
	MSC + Threshold	0.820 ± 0.010	0.785 ± 0.012	0.792 ± 0.011	0.220 ± 0.018
Deep Learning (on CSD)	DAGMM	0.895 ± 0.009	0.866 ± 0.010	0.869 ± 0.009	0.120 ± 0.012
	USAD	0.901 ± 0.008	0.874 ± 0.009	0.878 ± 0.008	0.115 ± 0.010
	OmniAnomaly	0.906 ± 0.007	0.880 ± 0.008	0.884 ± 0.008	0.095 ± 0.009
	DACR	0.919 ± 0.007	0.896 ± 0.007	0.895 ± 0.007	0.088 ± 0.008
	Anomaly Transformer	0.926 ± 0.006	0.901 ± 0.007	0.899 ± 0.006	0.082 ± 0.007
	TimesNet	0.925 ± 0.006	0.898 ± 0.006	0.902 ± 0.006	0.080 ± 0.006
	DCdetector	0.930 ± 0.005	0.905 ± 0.006	0.904 ± 0.005	0.075 ± 0.006
	TranAD	0.929 ± 0.005	0.903 ± 0.006	0.905 ± 0.005	0.074 ± 0.005
	KAN-AD	0.936 ± 0.005	0.907 ± 0.005	0.904 ± 0.005	0.070 ± 0.005
	CAROTS	0.941 ± 0.004	0.912 ± 0.005	0.910 ± 0.004	0.062 ± 0.004
**Ours**	**Physics-Gated**	**0.952 ± 0.002**	**0.921 ± 0.003**	**0.912 ± 0.002**	**0.048 ± 0.003**

**Table 7:** Architecture ablation results. A1 is the full proposed model; A2–A6 are variants with specific components removed or degraded. The best result in each column is in **bold**; ↓ indicates that lower values are better.

ID	Configuration	AUC-ROC	AUC-PR	F1	FPR@95%TPR ↓
A1	**Full Model** (CSD + VQ-VAE + Gated Fusion)	**0.952**	**0.921**	**0.912**	**0.048**
A2	CSD Only (no context branch)	0.891	0.854	0.862	0.112
A3	Context Only (no coupling branch)	0.873	0.837	0.843	0.135
A4	No channel expansion (original 4×4 CSD)	0.918	0.886	0.879	0.076
A5	Without synergy term ($D_{\mathrm{synergy}}$)	0.902	0.872	0.868	0.104
A6	Static fixed fusion (fixed $w_{1},w_{2}$)	0.915	0.884	0.882	0.088

**Table 8:** Pre-training strategy ablation results. C1 follows the complete training pipeline and is highlighted in bold as the proposed training strategy; C2–C4 skip one or both pre-training stages. The best result in each column is in **bold**; ↓ indicates that lower values are better.

ID	Training Strategy	AUC-ROC	AUC-PR	F1	FPR@95%TPR ↓
C1	**Full Pipeline** (Stage 1a → 1b → 2)	**0.952**	**0.921**	**0.912**	**0.048**
C2	No pre-training (direct end-to-end)	0.897	0.861	0.867	0.107
C3	CSD pre-trained only (skip VQ-VAE pre-training)	0.931	0.901	0.894	0.063
C4	VQ-VAE pre-trained only (skip CSD pre-training)	0.924	0.892	0.886	0.071

**Table 9:** Sensor durability evaluation over the entire continuous monitoring period.

Sensor Type	Evaluation Metric	Deployment Start	End of Monitoring Period
Pressure ($P_{1},P_{2}$)	Sensor Survival Rate	100%	100%
	Full-Scale Linearity	0.50%	0.52%
	Zero-Load Bias Offset	0.08% F.S.	0.14% F.S.
Vibration ($V_{1},V_{2}$)	Sensor Survival Rate	100%	100%
	Signal-to-Noise Ratio (SNR)	46.5 dB	43.2 dB
	Sensitivity Drift	Baseline (0%)	+1.35%

**Table 10:** Confusion matrix of the anomaly detection results on the test set, with an anomaly threshold calibrated at a 95% overall True Positive Rate.

True Condition	Total Samples	Predicted: Normal	Predicted: Anomaly
Normal (Various Regimes)	8687	8270	417 (FPR = 0.048)
Fault: Slipping	300	12	288 (TPR = 0.960)
Fault: Asymmetric Loading	300	18	282 (TPR = 0.940)
Fault: Mechanical Looseness	300	15	285 (TPR = 0.950)
Total Faults	900	45	855 (Overall TPR = 0.950)

## Data Availability

The data presented in this study are not publicly available due to confidentiality agreements with the industrial partner.
